# Prevalence of Central Airway Anatomical Variations and Structural Abnormalities Using Virtual Bronchoscopy: A Retrospective Study From a Tertiary Care Center

**DOI:** 10.7759/cureus.114126

**Published:** 2026-08-07

**Authors:** Jebasophi Prisilla, Haripriya M, Kalpana Ramachandran

**Affiliations:** 1 Anatomy, Sri Ramachandra Institute of Higher Education and Research, Chennai, IND

**Keywords:** anatomic variations, computed tomography (ct), structural abnormalities, tracheal diverticula, tracheobronchial tree, virtual bronchoscopy

## Abstract

Introduction

Virtual bronchoscopy (VB) is an imaging technique that does not involve surgery or other invasive procedures. It is an imaging technique that reconstructs a three-dimensional image of the airways from computed tomography (CT) scans. It has been found to be a possible substitute for conventional bronchoscopy to assess airway abnormalities without the drawbacks of invasive bronchoscopy. This study aimed to evaluate the feasibility of VB in detecting airway abnormalities and to establish the incidence of anatomical variations in the airways of a tertiary care centre.

Methods

A retrospective study evaluated adult patients who had undergone CT thorax over a period of six months. The airway abnormalities of the pulmonary CT were detected using Picture Archiving and Communication System (PACS). Patients under 18 years of age were excluded, along with those with poor-quality CT images. The GE ADW 4.7 workstation software was used to reconstruct the Digital Imaging and Communications in Medicine (DICOM) images into three-dimensional (3D) virtual bronchoscopic images. Patient demographics and airway abnormalities were analyzed using descriptive statistics.

Results

Of the 225 patients undergoing a CT scan of the thorax, 22 patients had abnormalities of the airways. Tracheal diverticulum was the most common abnormality, followed by tracheal stenosis and tracheomalacia. Other less common findings were tracheal bronchus, saber-sheath trachea, tracheoesophageal fistula, and Mounier-Kuhn syndrome. The airway anatomy was accurately reconstructed and detailed views of the site and size of the abnormalities could be performed using VB. It also showed promise for pre-procedural planning for assessment of the airway beyond stenotic segments.

Conclusions

CT-based VB is a valuable noninvasive imaging modality for evaluating central airway anatomy and identifying anatomic variations and structural abnormalities. It provides high-quality 3D visualization of the tracheobronchial tree, which may assist in airway assessment and pre-procedural planning. The findings of this study provide baseline data on the prevalence of central airway anatomic variations and structural abnormalities in patients from a tertiary care center and support the complementary role of VB in clinical practice.

## Introduction

The embryological development of the tracheobronchial tree forms the anatomical basis for understanding normal airway anatomy and the congenital variations encountered in clinical practice. The respiratory diverticulum arises from the ventral wall of the primitive foregut endoderm, caudal to the pharynx. The ventral wall rostral to the lung buds then pinches away from the dorsal wall to form a parallel tube. Caudal extension of this tube gives rise to the trachea anteriorly and the esophagus posteriorly. At about the end of the fourth week of development, the caudal end of the trachea bifurcates to give rise to left and right main bronchial buds, which grow into the adjacent layer of pleural mesenchyme derived from splanchnopleuric mesoderm [1].

The first step in branching morphogenesis is the formation of the first bronchial buds. The secondary bronchial buds - two on the left and three on the right - are formed by the asymmetrical division of the primary bronchial buds at the end of the fifth week. These buds will eventually give rise to the lobes of each lung.

The development of the last segment of the bronchopulmonary tree occurs during the maturation of the lungs during weeks five through seven; the somatopleuric and splanchnopleuric layers of mesoderm give rise to the parietal and visceral pleura of the lungs, respectively [1]. In order to shut the thoracic cavity and divide it from the abdominal cavity below, these pleuroperitoneal folds subsequently expand caudally and merge with the posterior margin of the septum transversum. The larynx, trachea, lung primordia, lung lobe, and bronchopulmonary segments all develop by the end of the embryonic period [1]. The main bronchi are formed by their quick division and branching.

The windpipe, also known as the trachea, is a cartilaginous tube that branches into the two main bronchi after extending from the larynx. It is necessary for air conduction to and from the lungs and develops from the caudal part of the laryngotracheal tube. Bronchial buds are the main outgrowths from the growing trachea, which will further split and differentiate to form the bronchial tree. Every bronchial bud will eventually develop into a main bronchus [2]. This phase represents the continuous development and branching of the bronchial buds into secondary and tertiary bronchi, further building the complicated network of airways inside the lungs. Significant cell growth and differentiation are involved in this process.

The laryngotracheal tube, surrounded by splanchnic mesoderm, emerges from the pharynx at the start of the fourth week of pregnancy, marking the beginning of the shown process. This is the first progenitor. After four weeks, there is a noticeable trachea and growing bronchial buds [2]. The trachea bifurcates as development proceeds, creating the major bronchi. These bronchial buds grow further, undergoing considerable branching and differentiation. Establishing the large surface area necessary for effective gas exchange in the adult lung depends on this complex arborization.

By eight weeks, the embryo clearly shows the identifiable lobar structure, with the lower lobe, middle lobe (on the right lung), and upper lobe all well-defined. This illustrates the amazing change from a simple tube to a complex, multilobed organ [2].

Phases of lobar bronchi development 

There are three developmental phases that occur throughout the bronchial tree. In phase one, the principal bronchus creates a symmetrical Y shape without any lobar swellings [3]. During phase two, although the structure is largely symmetrical, lobar swellings start to grow from the center of each bud, generating the left superior lobar bronchus and right middle lobar bronchus. In phase three, all the five unique lobar swellings result from the right superior lobar bronchus branching out. At this stage, there is notable asymmetry between the left and right principal bronchi [3].

Monopodial branching denotes the process wherein offspring branches arise directly from the sidewall of the parent bronchus. In their embryological analysis of the human bronchial tree, Fujii et al. reported that all lobar bronchi develop through monopodial branching. Additionally, nearly all of the 25 bifurcations that could be classified employ monopodial (or likely monopodial) branching when examined at the segmental and subsegmental levels. Dipodial branching forms additional branches, and the bronchus's tip splits in two. This modality was exceedingly infrequent in the human embryos studied; it was only detected at the bifurcation of the Lingular bronchus from the left superior lobar bronchus [3]. 

Following embryological development and branching morphogenesis, the tracheobronchial tree assumes its definitive anatomical configuration. The trachea is a midline fibrocartilaginous airway extending from the lower border of the cricoid cartilage at the level of the C6 vertebra to its bifurcation at the upper border of the T5 vertebra, where it divides into the right and left main bronchi. Tracheal dimensions exhibit physiological variations according to age, sex, and body habitus, and morphometric values reported in the literature show some variability among anatomical studies [4].

Direct visualization of the airways is essential for the diagnosis and evaluation of airway abnormalities. Alfred Kirststein first visualized the proximal airway using a modified esophagoscope. Subsequently, Gustav Killian pioneered bronchoscopy by using a laryngoscope to visualize the main bronchi and successfully remove foreign bodies from the respiratory tract. Later, Chevalier Jackson introduced significant improvements to the bronchoscope, including enhanced illumination and suction, which greatly improved the safety and effectiveness of rigid bronchoscopy [5].

Flexible bronchoscopy was introduced by Shigeto Iketa, a world-renowned thoracic surgeon. Flexible bronchoscopy, which has been available since 1968 is regarded as the second revolution of bronchoscopies. Hereafter, several significant modifications and developments of the current bronchoscopy, which is already used in clinical practice, were made [5]. Currently, both diagnostic and therapeutic bronchoscopy exist.

The diagnostic applications of bronchoscopy include assessment of airway anatomy, evaluation of airway dynamics, and collection of tissue and fluid samples for diagnostic analysis. It aids to diagnose airway abnormalities such as accessory, supernumerary or absent airway, tracheomalacia, stenosis, tracheobronchial or vascular malformations, extrinsic airway compression and to obtain specimen to evaluate infections or malignancies. Clinically, it is employed at the time of removal of blood/mucous plugs, foreign body removal or surgery for removal of airway masses, airway leak management, stenting or airway dilatation procedures for relief of stenosis [6].

Airway abnormalities comprise a diverse group of congenital and acquired conditions with distinct anatomical and clinical implications. Congenital anomalies, including tracheal bronchus, accessory cardiac bronchus, congenital tracheoesophageal fistula, and Mounier-Kuhn syndrome, may alter normal tracheobronchial anatomy and predispose patients to recurrent respiratory infections, airway obstruction, or other pulmonary complications. Acquired abnormalities, such as tracheal stenosis, tracheobronchopathia osteochondroplastica, saber-sheath trachea, tracheomalacia, and tracheal diverticulum, may further compromise airway function and present diagnostic and therapeutic challenges. Accurate recognition of these abnormalities is essential for bronchoscopy, endotracheal intubation, thoracic surgery, and interventional airway procedures, where unrecognized anatomical variations may result in procedural difficulties or complications [7-9]. 

Bronchoscopy can also help in diagnosis and treatment, but has its own complications. It can be minor, major or sometimes fatal. There are complications associated with the procedure or with the sedation medications that are administered. A few notable complications include trauma, haemorrhage, infection, bronchospasm and atelectasis [10]. Elderly, frail, or medically compromised patients may not be suitable candidates for conventional bronchoscopy because of its invasive nature. Nevertheless, bronchoscopy remains the gold standard for the diagnosis and evaluation of a wide range of airway disorders. Recent advances in three-dimensional (3D) reconstruction of computed tomography (CT) images have facilitated the development of virtual bronchoscopy (VB), a noninvasive imaging technique that provides endoluminal visualization of the tracheobronchial tree. VB uses high-resolution CT datasets to generate 3D endoluminal reconstructions of the tracheobronchial tree. It enables visualization of airway lesions, assessment of their extent, evaluation of distal airway patency beyond stenotic segments, and facilitates pre-procedural planning for diagnostic and therapeutic interventions. In addition, VB provides valuable anatomical information in conditions such as Mounier-Kuhn syndrome, congenital tracheoesophageal fistula, and saber-sheath trachea, and serves as an effective tool for medical education and procedural simulation. However, it cannot assess dynamic airway changes, making it less suitable for disorders such as tracheomalacia, and image quality may be compromised by respiratory or patient motion artifacts. Furthermore, its inability to perform tissue sampling or therapeutic interventions and its limited ability to detect subtle mucosal abnormalities or very small airway openings preclude it from replacing conventional bronchoscopy. Consequently, VB is best considered a complementary imaging modality that enhances, rather than replaces, conventional bronchoscopy in the evaluation of central airway disorders [7-9,11].

Despite these limitations, VB has emerged as a valuable complementary imaging modality for airway evaluation. While several studies have compared the diagnostic performance of VB with conventional bronchoscopy, most have focused on diagnostic accuracy or specific airway pathologies rather than providing comprehensive epidemiological data on tracheobronchial anatomical variations. Accurate identification of these variations is clinically important for bronchoscopy, endotracheal intubation, thoracic surgery, and other airway interventions, as unrecognized anomalies may lead to diagnostic errors or procedural complications [10,11]. Therefore, the present study was undertaken to determine the prevalence of central airway anatomic variations and structural abnormalities using CT-based VB in patients from a tertiary care center, thereby contributing baseline data that may aid clinical practice and future research.

## Materials and methods

This retrospective, observational, imaging-based study was conducted at the Department of Anatomy in collaboration with the Department of Radiology. The study period extended from July 2024 to December 2024. Ethical approval was obtained from the Institutional Ethics Committee of Sri Ramachandra Institute of Higher Education and Research (approval no. CSP-MEDI24/AUG/107/269). As this was a retrospective study utilizing anonymized imaging data, the requirement for informed consent was waived.

Study population

CT-VB images of adult patients who underwent VB evaluation during the study period were included. The majority of patients were referred for evaluation of suspected or established conditions.

Inclusion Criteria

This retrospective study included only patients aged 18 years or older who had undergone CT chest examinations demonstrating central airway abnormalities that were suitable for VB reconstruction. 

Exclusion Criteria

CT scans with artifacts or poor image quality that precluded accurate VB reconstruction were excluded.

Demographic data

CT acquisition and reconstruction parameters are important determinants of VB image quality. Accordingly, we have included details of the CT acquisition protocol, including the scanner model, contrast protocol, slice thickness, reconstruction algorithm, and image reconstruction/window settings used for VB. The CT Digital Imaging and Communications in Medicine (DICOM) images were transferred to the GE Advantage Workstation (ADW) version 4.7 (GE HealthCare Technologies Inc., Chicago, Illinois, USA), where 3D segmentation and VB reconstruction were performed. These were considered VB images for abnormalities. The main aim was to examine the feasibility of VB in the patients with abnormal airway findings as seen in the CT chest. The secondary aims were to determine the prevalence of the different airway anatomical variations in the listed study period. As this was a retrospective study, a sample size was not calculated. Median, range, and percentages were used to depict patient characteristics. Descriptive statistics were used to depict airway abnormalities. The ethics committee of the institution approved the study. Patients' informed consent was waived off since it was a retrospective study.

## Results

**Figure 1:**
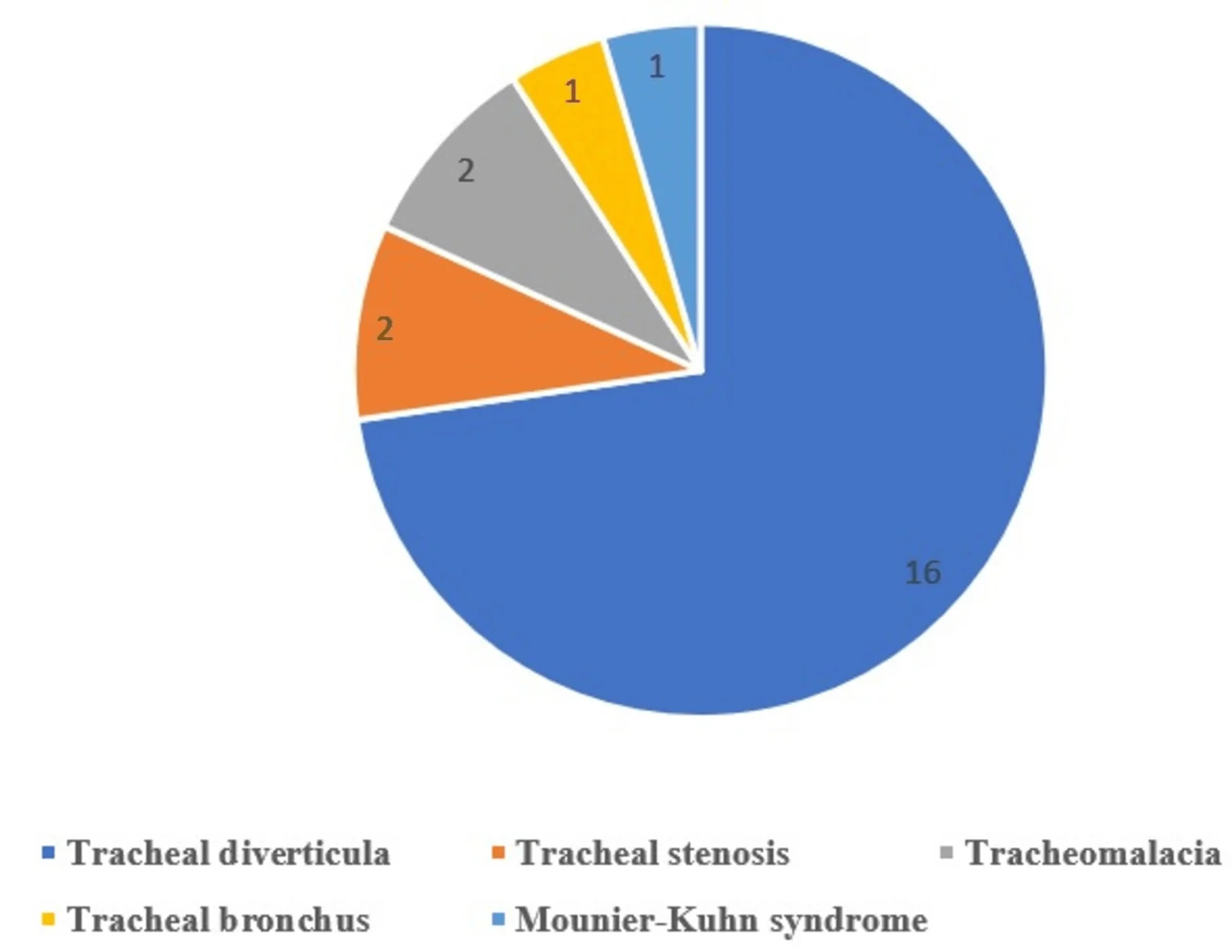
Pie chart illustrating the distribution of cases among the various tracheobronchial anomalies

A total of 284 thoracic CT examinations were retrospectively reviewed. Twenty-two patients were identified as having abnormal findings on the CT airways. Figure 1 shows various anomalies identified with their respective incidences.

**Figure 2:**
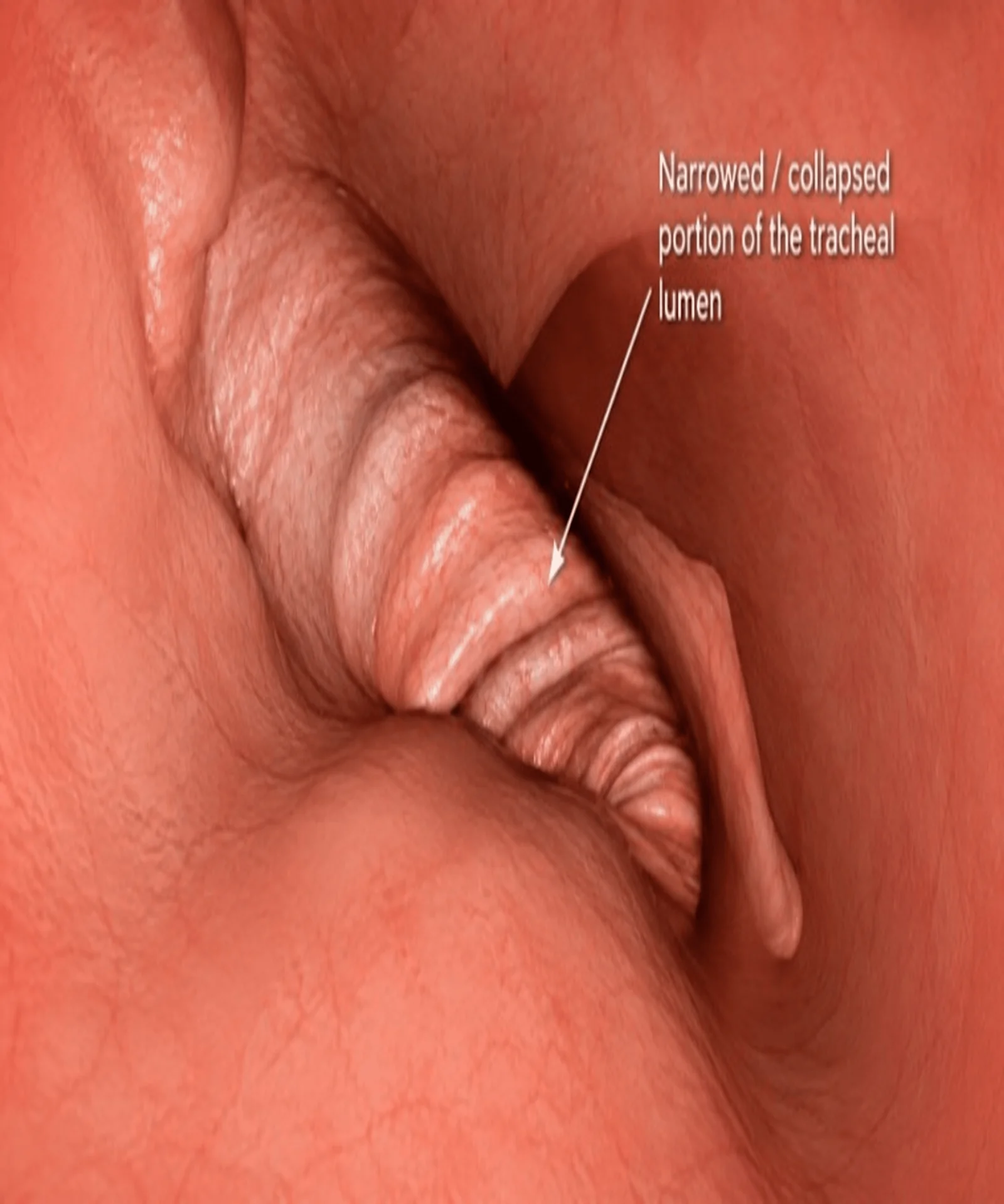
Tracheomalacia (dynamic tracheal airway collapse)

Figure 2 indicates the narrowed/collapsed tracheal lumen caused by dynamic airway collapse during expiration.

**Figure 3:**
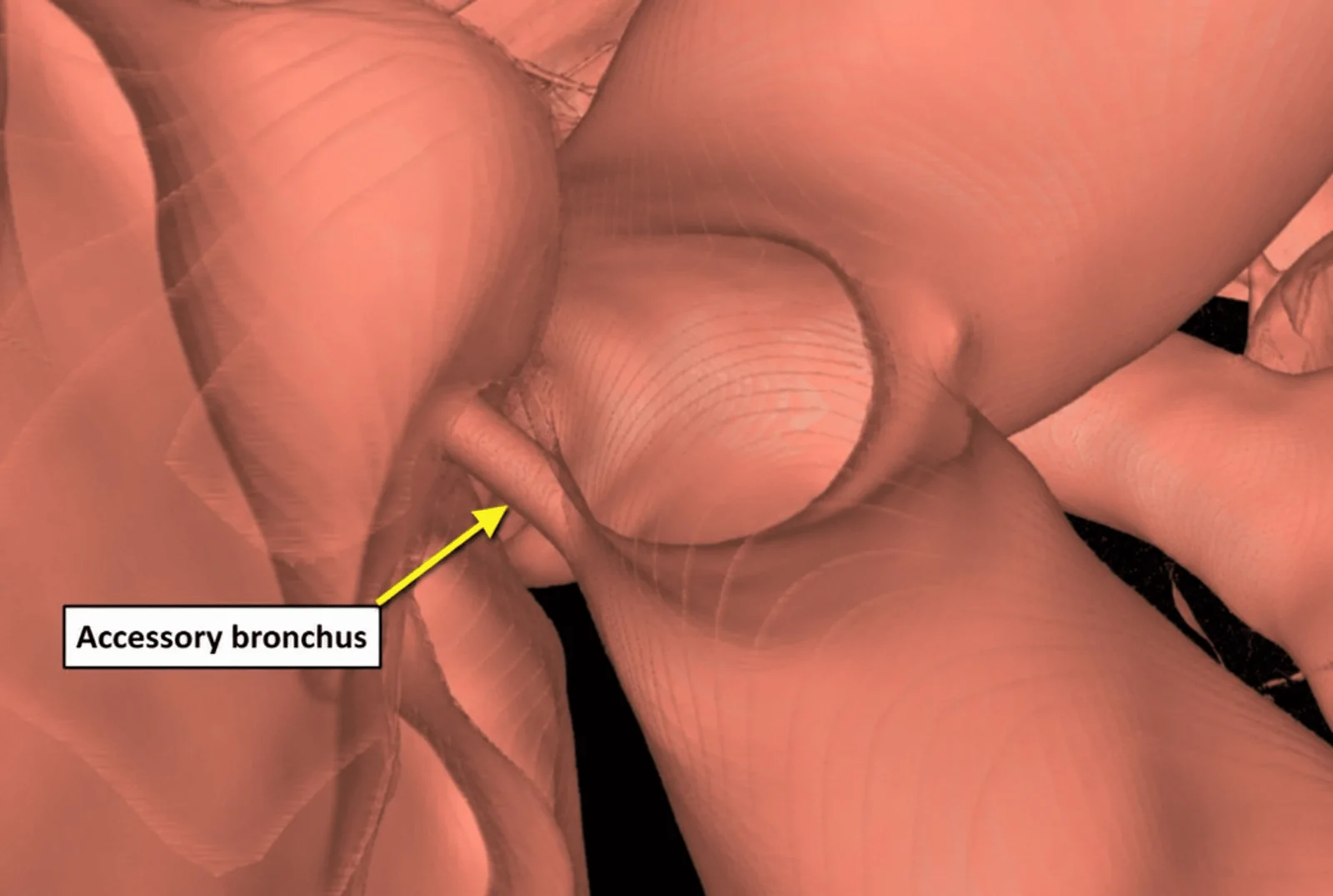
Accessory bronchus

A 3D reconstructed image demonstrating an accessory bronchus is shown in Figure 3.

The arrow indicates a supernumerary bronchial branch arising from the tracheobronchial tree, representing an anatomical variation of the bronchial system. 

**Figure 4:**
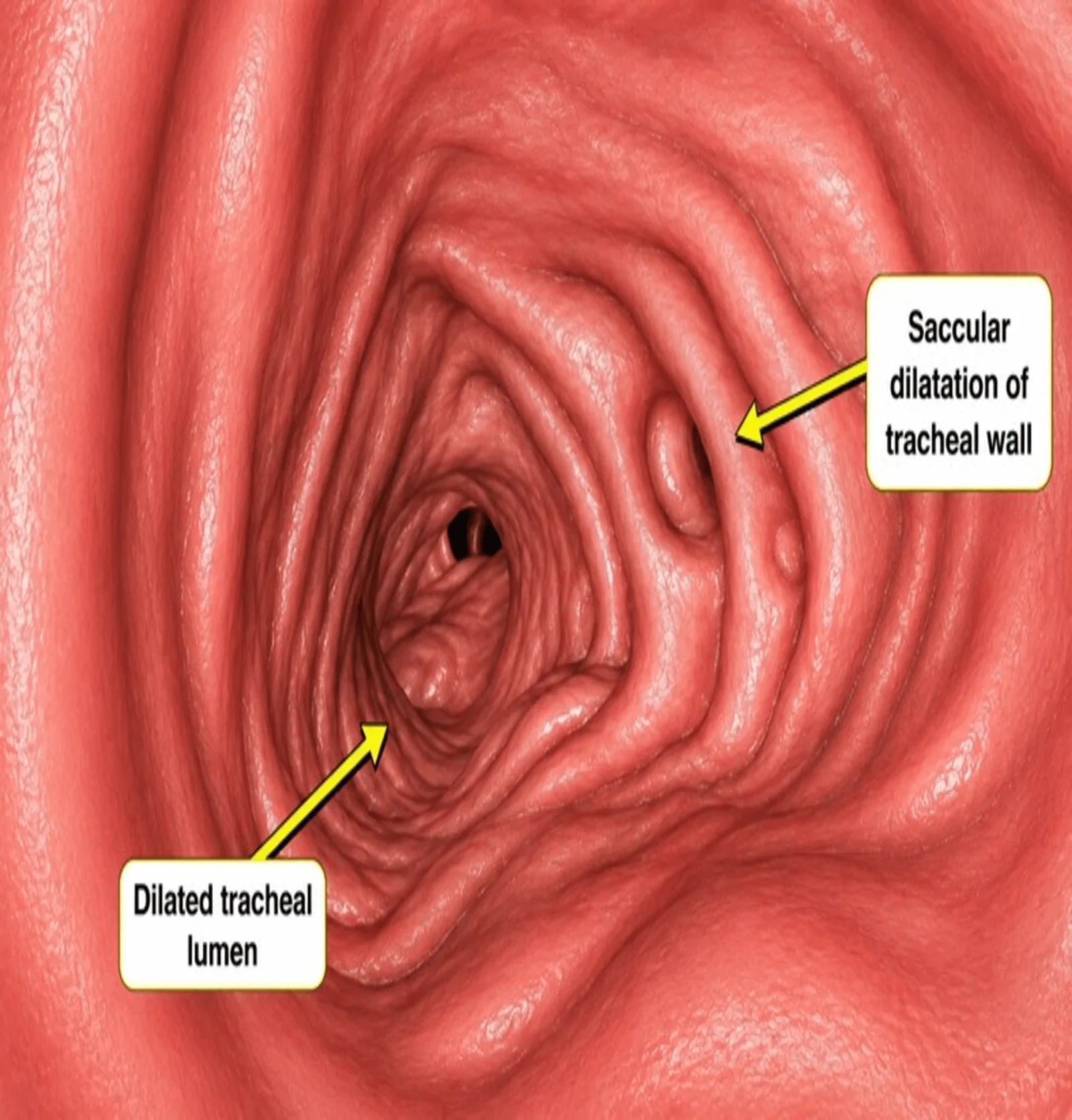
Mounier–Kuhn syndrome (tracheobronchomegaly)

A 3D endoluminal reconstructed image demonstrating the characteristic features of Mounier-Kuhn syndrome is shown in Figure 4. 

The arrow indicates saccular dilatation of the tracheal wall, while the second arrow indicates the dilated tracheal lumen, reflecting marked tracheobronchial enlargement due to atrophy of the elastic and smooth muscle components of the tracheobronchial wall. 

**Figure 5:**
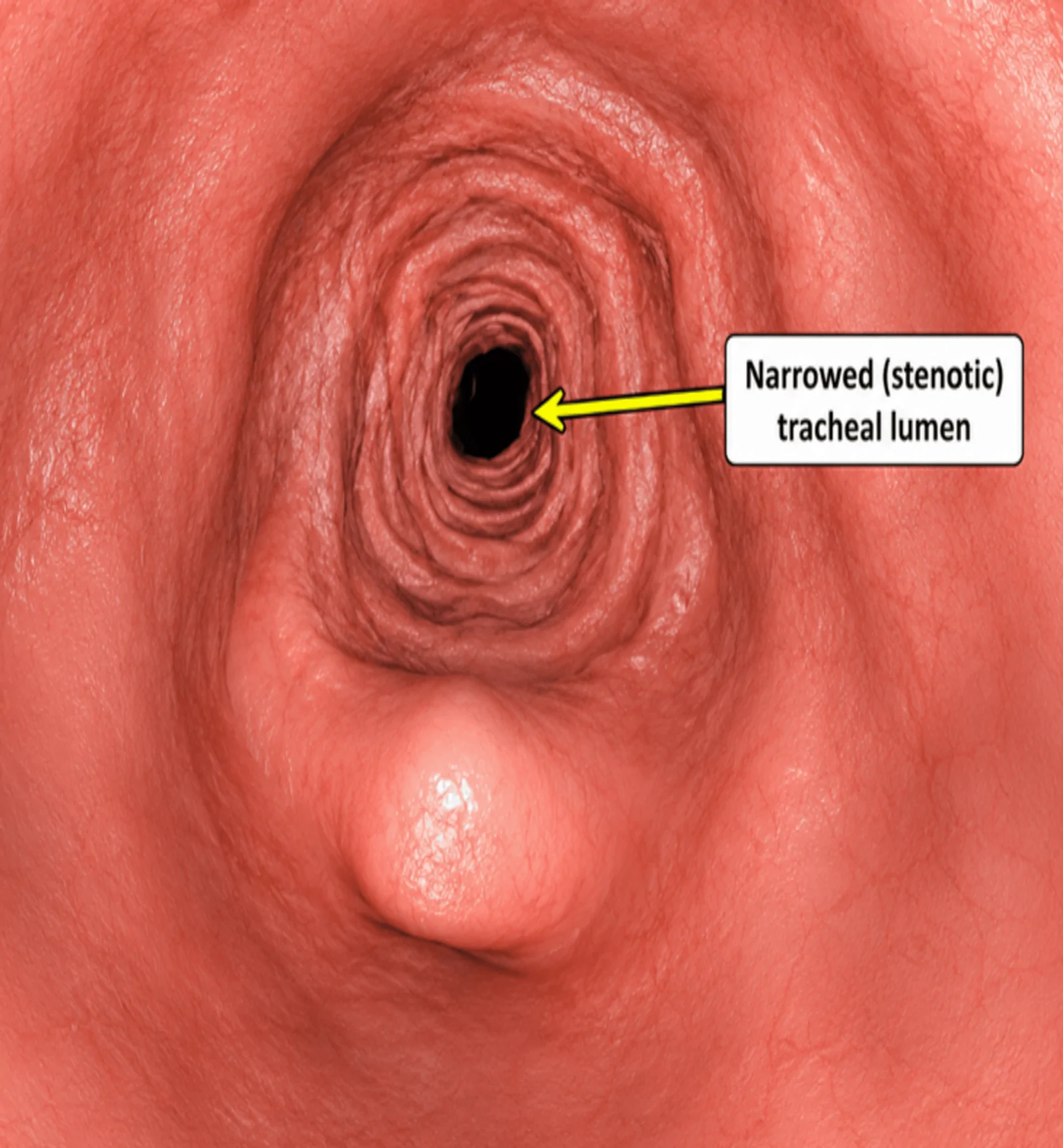
Tracheal stenosis

Figure 5 denotes a 3D endoluminal reconstructed image demonstrating tracheal stenosis.

The arrow indicates the focal narrowing of the tracheal lumen caused by circumferential reduction in airway diameter, resulting in restricted airflow through the affected segment. 

**Figure 6:**
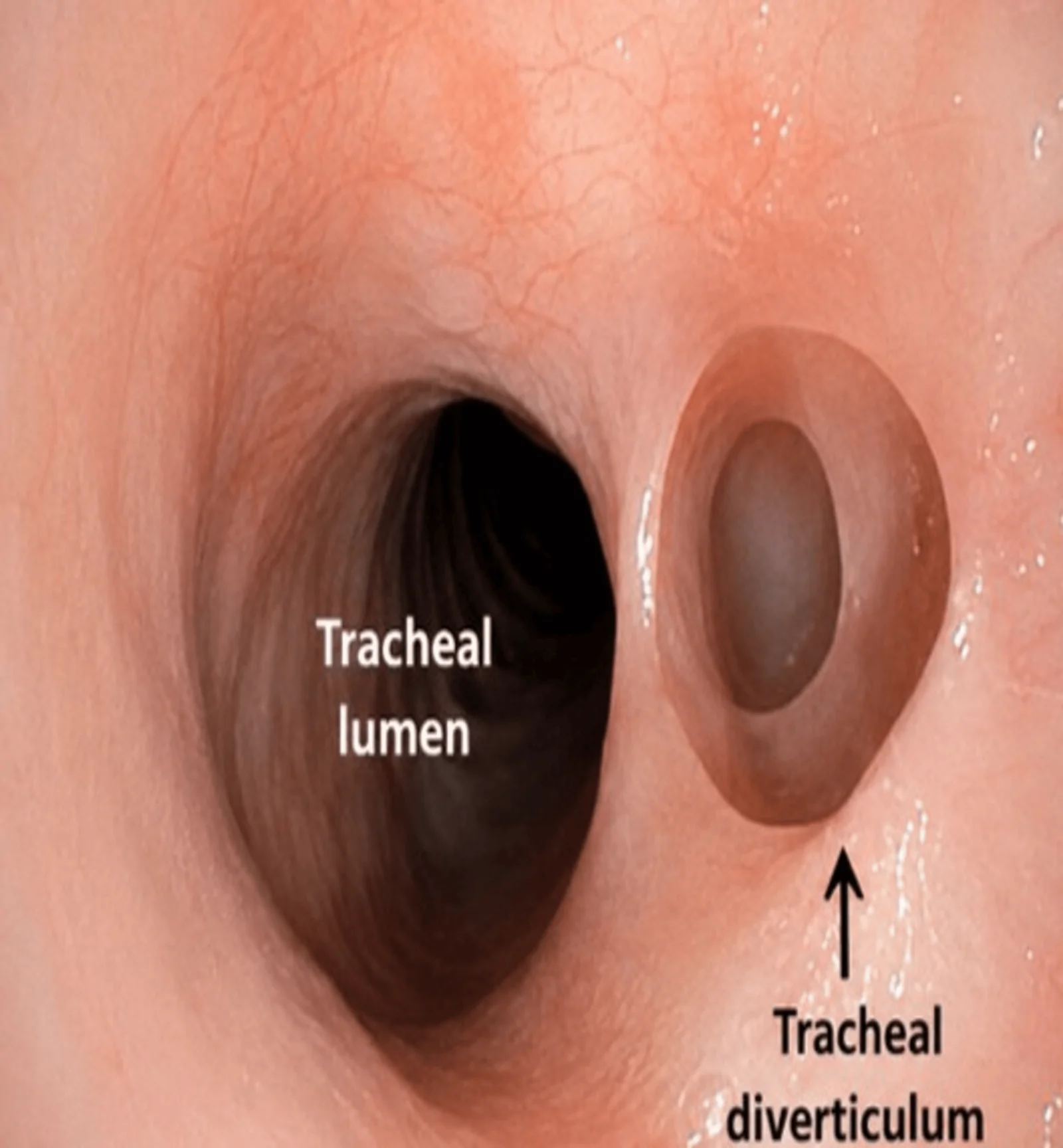
Tracheal diverticulum

Figure 6 is a VB image demonstrating a tracheal diverticulum arising from the right posterolateral wall of the trachea.

The central tracheal lumen is visualized, and the diverticular opening is indicated by the arrow.

## Discussion

Post-intubation injury and inflammation, infection, trauma, and/or neoplasm can cause tracheal stenosis [12]. Tracheal bronchus is classified into five types based on the existence of a right upper lobe bronchus. Most of the time, it is associated only with recurrent cough and bronchitis and is not a significant problem [13]. Tracheomalacia may be either congenital or acquired. Congenital causes include malformation of the cartilage rings, leading to excessive collapsibility of the trachea. Acquired tracheomalacia is caused by a compromise of the rings in the trachea. The causes are related to traumatic intubation or to surgical injury of the thoracic cavity during surgery. There is also a suggestion of damage over a prolonged period resulting from irritants such as smoking [14,15]. In saber-sheath trachea, the anteroposterior diameter is broadened, while the lateral diameter is shortened. Although very uncommon, it often occurs in chronic obstructive pulmonary disease (COPD). It is typically discovered incidentally during imaging [16].

Direct bronchoscopy is the gold standard procedure to diagnose as well as intervene in airway abnormalities. It is possible with either fiberoptic or rigid bronchoscopy. For smaller airway assessment, fiber optics are ideal, and central airway interventions such as stenting are better with rigid bronchoscopy. Fiberoptic bronchoscopy (FOB) is done under local anesthesia with or without intravenous sedation. Rigid bronchoscopy requires general anesthesia for patient comfort. Though bronchoscopy is considered a safe intervention, it can still lead to major complications in around 4.3% of patients. In frail patients, the procedure of direct bronchoscopy is difficult [17]. However, direct bronchoscopy remains the standard technique for performing bronchoscopic procedures such as tissue sampling, lavage, and stenting.

VB is the noninvasive alternative to conventional FOB in evaluating airway abnormalities such as tracheal airway stenosis, tracheomalacia, and other anomalies derived from the CT multidetector scan. Further, it can help to preplan a bronchoscopy procedure. For example, in the prospective Nateglinide And Valsartan in Impaired Glucose Tolerance Outcomes Research (NAVIGATOR) study, VB was used to plan access to small pulmonary nodules. The diagnostic yield was as high as 77% with VB-based preplanning. Therefore, the standard bronchoscopy/VB navigation system is very useful for producing higher yields in the case of a challenging crop. Our study results show high feasibility in some airway abnormalities and a good diagnostic accuracy. Overall, this is consistent with VB's capacity to yield 3D views of the endoluminal surfaces, with none of the sedation, bleeding, or infection risks imposed by FOB. Additionally, it can help as a pre-planning device prior to FOB for determining the severity of the abnormality, caliber of the airway beyond the abnormality, and sites for biopsy. VB can help identify the site, length, and degree of stenosis.

The main benefit of using VB is its ability to evaluate distal airway patency beyond the stenosis. In the retrospective study performed by Hoppe et al., the accuracy of VB in determining the presence of tracheal stenosis (98%) was greater than that of the reformatted CT images. In addition, grading of tracheal stenosis was more accurate in comparison to reformatted CT images. Except for diagnostic or therapeutic procedures, however, direct bronchoscopy remains the gold standard [18]. VB can give important diagnostic indications and can assist with diagnostic procedures.

The tracheal bronchus is characterized by the anomalous origin of the bronchus from the trachea. There are several theories regarding the origin of the tracheal bronchus. Reduction theory assumes the presence of a primitive pattern of tracheobronchial branching, which was later to be reduced to form the final architecture [19]. According to migration theory, in the initial phase a bilateral symmetric branching pattern exists, and then subsidiary branches can migrate to a different location within the tracheobronchial tree [20]. Based on selection theory, tracheal bronchus develops when bronchial mesenchyme contacts tracheal epithelium [21]. However, routine usage of VB in tracheomalacia is not recommended since it cannot show dynamic airway changes. VB had a moderate sensitivity and good specificity in a pediatric population [22].

In adults, tracheoesophageal fistula (TEF) is most often a result of malignancy, with the rest due to benign conditions such as surgical injuries. There are several advantages of VB in the assessment of TEF. It is non-invasive; thus, it helps to alleviate the risk of infection/perforation. It can also be used to plan surgery and for diagnosis of airway collapse. VB is a valuable aid to the diagnosis in some forms of congenital TEF, particularly those of the H type [9]. On the other hand, biopsy is crucial for diagnoses and treatment of malignancies, which cannot be performed by VB. It leads to recurring respiratory infections [23]. In most of the cases, it is associated with tracheal diverticula, which may be acquired or congenital. The exact etiology of this syndrome is unknown, though genetic susceptibility is postulated [24]. Clinically, it is asymptomatic, or can manifest as recurrent respiratory tract infections. VB can help to identify the region of dilatation and any associated tracheal diverticula. Mounier-Kuhn syndrome is characterized by tracheal dilatation due to thinning of the tracheal wall [25]. Anatomical airway abnormalities were the most detected in our study, with the majority being tracheal diverticula. It may be congenital or developed later in life. Most of the diverticula do not give rise to symptoms and are discovered accidentally during evaluation. Although VB can identify diverticula, it still cannot identify a diverticulum with a narrow opening. CT with very thin slice thickness is still leading the way with regard to the diagnosis of TC. However, there are some problems in reconstruction, such as patients not being cooperative, as this leads to reduced image quality due to motion artifacts. VB cannot evaluate changes in the airway's size and can't take biopsies; thus, there is a need for combining VB with FOB.

The retrospective nature of the study and the associated biases are the major limitations. In addition, the sample size and design of one or a few institutions may not represent the true real-world data and therefore be misrepresentative of the prevalence. The study didn’t compare the findings of VB and direct bronchoscopy or the advantages of VB prior to a direct bronchoscopy, which is crucial to establish the role of VB in clinical situations. Also, our study population consisted of an adult patient cohort. As a result, no data on pediatric congenital airway anomalies were evaluated.

Strengths

The study showed good clinical feasibility, as the present study was designed to evaluate the prevalence of central airway anatomic variations and structural abnormalities using CT-based VB and not to assess its diagnostic accuracy against conventional bronchoscopy or another gold standard, the detection of airway abnormalities, with the tracheal diverticula being the most common anatomic variant identified in its cohort. The study identified VB as a safe, noninvasive approach that sidesteps the risks of sedation, bleeding, and infection associated with conventional FOB. The ethical application of a GE ADW4.7 workstation to produce detailed 3D reconstructions from anonymized CT images was mentioned. Furthermore, the data also demonstrated the important pre-procedural value of VB in assessing the degree of abnormalities and the extent of distal airways beyond stenotic regions before physical interventions.

Limitations

This study has several limitations. First, it was a retrospective, single-center study, which may limit the generalizability of the findings. Second, VB findings were not directly validated against conventional bronchoscopy in all patients because bronchoscopic evaluation was not clinically indicated or available for every case. Therefore, the diagnostic accuracy of VB could not be determined. Third, VB cannot evaluate dynamic airway changes, subtle mucosal abnormalities, or perform tissue sampling and therapeutic interventions. In addition, respiratory or patient motion may introduce image artifacts that can affect the accuracy of 3D reconstructions. Future prospective, multicenter studies with direct comparison between VB and conventional bronchoscopy are warranted to further establish the diagnostic value of VB in the assessment of central airway abnormalities.

## Conclusions

CT-based VB is a valuable noninvasive imaging modality for evaluating central airway anatomy and identifying anatomical variations and structural abnormalities. It provides detailed three-dimensional visualization of the tracheobronchial tree, facilitating accurate airway assessment and pre-procedural planning. The findings of this study provide important epidemiological data on the prevalence and spectrum of central airway variations and abnormalities in patients from a tertiary care center. Early recognition of these anatomical variations is essential for bronchoscopy, endotracheal intubation, thoracic surgery, and other airway interventions to minimize procedural complications. Although VB does not replace conventional bronchoscopy, it serves as a valuable complementary imaging tool in the evaluation of central airway disorders. Further prospective multicenter studies are warranted to validate these findings and expand the clinical applications of VB.
